# The effect of flap advancement on bone graft displacement with or without membrane stabilization – a preclinical study

**DOI:** 10.1186/s12903-025-06692-x

**Published:** 2025-08-11

**Authors:** Wenjie Zhou, Emilio Couso-Queiruga, Emilio A. Cafferata, Ausra Ramanauskaite, Frank Schwarz, Clemens Raabe

**Affiliations:** 1 Department of Oral Surgery and Implantology, Goethe University, Carolinum, Frankfurt am Main, Germany; 2 Second Dental Center, School of Medicine, Ninth People’s Hospital, Shanghai Jiao Tong University, Shanghai, China; 3 Department of Oral Surgery and Stomatology, School of Dental Medicine, University of Bern, Bern, Switzerland; 4 Oral Peri-Implant Research Group, School of Dentistry, Universidad Científica del Sur, Lima, Perú

**Keywords:** Alveolar ridge augmentation, Cone-beam computed tomography, Dental implants, Guided bone regeneration, Surgical flaps, Bone substitutes, Flap advancement, Flap tension, Membrane fixation, Primary wound closure

## Abstract

**Background:**

Displacement of bone graft materials following horizontal bone augmentation (HBA) procedures may compromise treatment outcomes, yet the role of flap tension remains unclear. This study aims to assess the effect of varying degrees of flap advancement (FA) and associated flap tension on graft displacement during HBA, as well as the influence of membrane stabilization and soft tissue characteristics.

**Methods:**

HBA was performed at the bone concavity of the diastema region in pig hemimandibles, corresponding to uncontained defect morphologies. Full-thickness mucoperiosteal flaps were coronally advanced using a single periosteal releasing incision (moderate FA1) or additional scoring (major FA2). Sites were randomized for membrane fixation with two pins or no fixation. Changes in graft material thickness (ΔGMT) were assessed at nine reference levels (L0–L8) before and after wound closure via CBCT. Linear regression models were applied to correlate ΔGMT with the FA, fixation and soft tissue phenotypical characteristics.

**Results:**

Sixty surgical procedures were categorized into four groups (FA1 - Pins, FA1 + Pins, FA2 - Pins, FA2 + Pins). FA2 achieved greater advancement (9.20 ± 0.55 mm) with lower tension (0.02 ± 0.01 N) than FA1 (4.42 ± 0.67 mm; 0.09 ± 0.02 N). Reduced graft displacement was observed at L0 with a mean ΔGMT of -35.66 ± 30.68% for FA2 versus − 44.99 ± 20.80% for FA1 (*p* < 0.001). Membrane stabilization did not significantly influence the overall ΔGMTs. Regression analysis revealed a significant correlation between FA and ΔGMT (*p* = 0.01), independent of the fixation method (*p* = 0.56), and soft tissue phenotypical characteristics (*p* > 0.06).

**Conclusions:**

Greater flap advancement and reduced tension improve graft stability during HBA, while membrane fixation with two pins is insufficient to ensure graft stability in challenging, uncontained defect morphologies.

## Background

Tooth loss initiates a cascade of wound healing and remodeling processes that result in local soft and hard tissue changes. These events often lead to horizontal and/or vertical alveolar ridge deficiencies, which pose significant challenges to tooth replacement therapy via dental implants [1–3]. To restore adequate alveolar ridge dimensions suitable for dental implant placement, horizontal bone augmentation (HBA) is typically performed using particulate bone grafting materials covered by a resorbable collagen membrane, following the principles of guided bone regeneration (GBR). This approach is widely adopted in clinical practice due to its ease of handling, low complication rates and long-term success [4–6]. However, despite nearly 40 years of refinement, HBA remains highly dependent on surgical skills and experience, making consistent and predictable outcomes in some clinical scenarios still challenging [7].

Achieving primary wound closure is an essential prerequisite for successful graft material integration during tissue regeneration procedures, as it protects the graft from exposure to the oral environment [8]. To obtain passive, and tension-free closure in HBA, various flap management strategies have been proposed, including full-thickness mucoperiosteal or combination flaps with various designs [9–12]. Among these, full-thickness mucoperiosteal flaps are the most commonly employed, often accompanied by periosteal-releasing incisions to allow adequate flap advancement [13, 14]. According to Greenstein and collaborators, flap advancement can be classified as minor (< 3 mm), moderate (3–6 mm), and major (≥ 7 mm), with major advancements typically requiring deeper periosteal scoring (3–5 mm into the submucosa) [11, 15].

Inadequate flap advancement leading to high flap tension can compromise primary wound closure, and negatively affect HBA outcomes. Similarly, excessive flap tension is associated with an increased risk of complications, such as wound dehiscence, membrane exposure, and graft loss [16]. Furthermore, post-operative wound exposure and contamination have been significantly associated with a marked reduction in bone formation, by as much as five- to six-fold, potentially resulting in adverse healing or even complete treatment failure [17].

Most studies assessing the effectiveness of bone augmentation procedures rely on immediate postoperative measurements as the baseline, without accounting for potential graft material displacement caused by flap manipulation and primary wound closure [5]. Indeed, recent studies have highlighted the collapse of particulate grafts and collagen membranes during wound closure, leading to suboptimal graft dimensions [18, 19]. To mitigate these dimensional changes, the use of bone pin fixation or periosteal suturing has been proposed to stabilize graft material [18–22]. However, these methods are technically demanding and time-consuming, and their efficacy in uncontained defects remains insufficiently documented [19]. Additionally, the need for collagen membrane fixation in HBA procedures is still controversial, as current evidence remains inconclusive [23].

Therefore, the present study aimed to evaluate the effects of two different flap advancements, corresponding flap tensions, and membrane stabilization on the dimensional changes of graft materials during HBA in uncontained defect morphologies. The null hypothesis stated that neither flap advancement (H01), membrane fixation (H02), nor soft tissue phenotypical characteristics (H03) would significantly influence bone graft dimensional stability following primary wound closure.

## Methods

### Preclinical models and study groups

This investigation was carried out at the Department of Oral Surgery and Implantology, Goethe University, Carolinum, Frankfurt am Main, Germany, between June and October 2024. Ethical approval was not required, as this study involved the use of fresh hemimandibles from 30-week-old pigs, sourced from a local butcher.

HBA procedures were performed sequentially, first with a moderate amount of flap advancement, followed by a major flap advancement. The use of pins for membrane fixation was randomly assigned for each hemimandible. This design yielded four equally sized study groups:

- FA1 - Pins: Moderate flap advancement without pins.
- FA1 + Pins: Moderate flap advancement with pins.
- FA2 - Pins: Major flap advancement without pins.
- FA2 + Pins: Major flap advancement with pins.

### Surgical procedure

HBA procedures via GBR were performed at the bony concavity of the diastema region in pig hemimandibles, simulating a horizontal alveolar ridge deficiency with an uncontained defect morphology. All procedures up to graft material placement, including membrane positioning and, if allocated, pin fixation, were performed by a single board-certified and experienced oral surgeon (CR). Subsequently, a second operator (EAC), who was blinded to the study design and objectives, performed the primary wound closure.

Preoperatively, site-specific soft tissue phenotypic characteristics at the region of interest were recorded. These included facial keratinized mucosa width (KMW) measured with a PCPUNC15 probe (Hu-Friedy, Chicago, Illinois, United States), and facial mucosal thickness (MT) with an endodontic file (Finger Spreader ISO 20, Dentsply Sirona, Charlotte, North Carolina, United States) through horizontal transmucosal probing at 3, 6, and 9 mm (MT3, MT6, MT9) apical from the mucosal margin.

**Fig. 1:**
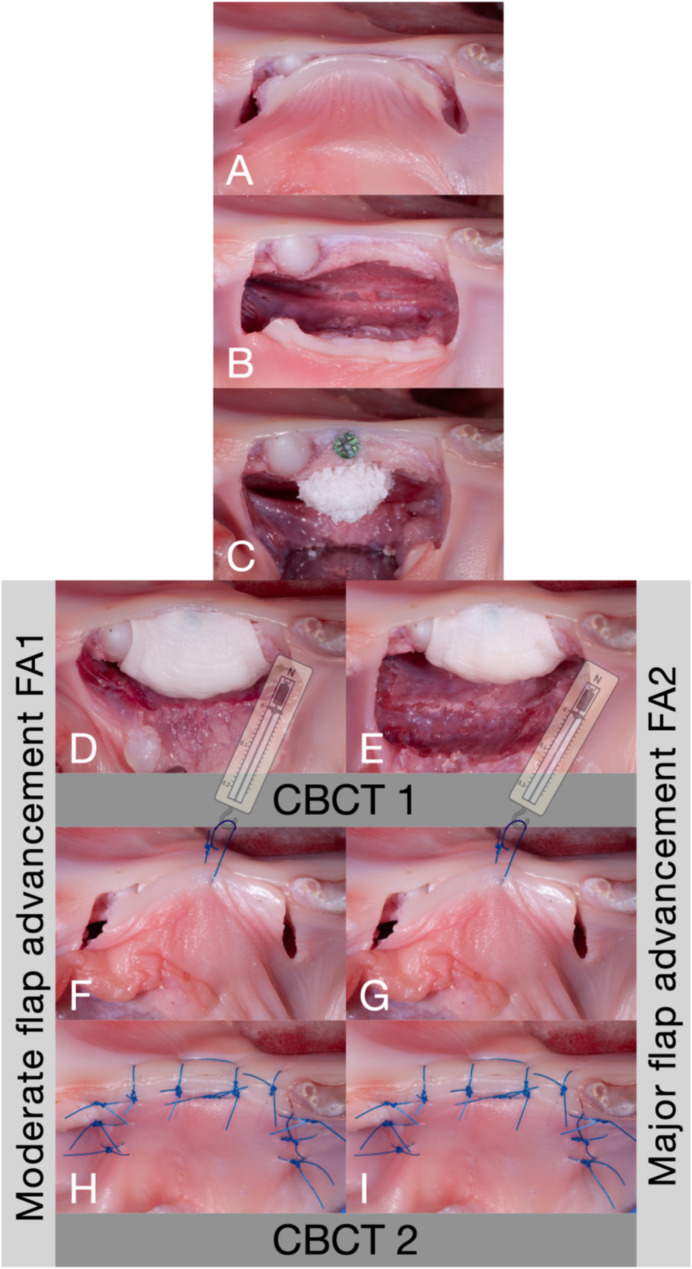
A standardized trapezoidal incision line was created in the diastema region of the mandibles (**A**). The buccal flap was elevated by bluntly detaching the periosteum from the bone, reaching the apical level of the vertical releasing incisions (**B**). A titanium screw was centrally placed in the ridge, followed by the augmentation of the bone concavity in the diastema region with particulate bone graft material (**C**). A collagen membrane was placed to cover the bone graft and the sites were randomized for membrane fixation using two pins. A periosteal releasing incision was made at the base of the flap, aiming at a moderate flap advancement of 4–5 mm (**D**, FA1), or additional stretching was carried out to achieve a major flap advancement of 9–10 mm (**E**, FA2) and an intraoperative cone-beam computed tomography (CBCT1) was taken. In both FA1 and FA2, the flap tension was assessed using a spring dynamometer attached to a central single-interrupted suture (**F**/**G**). Primary wound closure was then performed using one horizontal mattress suture and nine single-interrupted sutures (**H**/**I**). After wound closure, the mandible was scanned again (CBCT2)

A standardized trapezoidal incision line was marked on the mucosa using a foil template: a 20 mm crestal incision with two 10° diverging vertical releasing incisions, each 10 mm in length (Fig. 1). The buccal full-thickness flap was elevated by bluntly detaching the periosteum with a raspatory, extending apically to the end of the vertical releasing incisions. A mid-crestal osteotomy was performed, and an osteosynthesis screw (diameter 1.4 mm, length 11 mm, Micro-Screw, Ustomed, Tuttlingen, Germany) was centrally positioned at the defect site to serve as a radiographic reference marker for standardized measurement.

#### Moderate flap advancement (FA1)

A sharp, perpendicular periosteal incision (≈ 0.5 mm depth) was made at the base of the flap. The tissues were then stretched along the incision line using a blunt raspatory in a sweeping motion, achieving 4–5 mm of flap advancement. The defect was augmented using particulate demineralized bovine bone material (Bio-Oss^®^, Geistlich AG, Wohlhusen, Switzerland) pre-soaked in a radiopaque contrast agent (Gastrolux, Sanochemia Pharmazeutika, Neufeld an der Leitha, Austria). The graft was covered with a collagen membrane (Bio-Gide^®^, Geistlich AG, Wohlhusen, Switzerland) targeting a horizontal gain of 3 mm. If allocated, the collagen membrane was stabilized using two non-threaded press-fit pins (Frios Plus, Dentsply Sirona, Charlotte, North Carolina, United States), placed bucco-apically in a standardized, perpendicular orientation with an inter-pin distance of 15 mm. Following HBA, an intraoperative CBCT scan was acquired (8 × 8 cm, 80 kV, 6 mAs, PaX-Reve3D, Vatech, Hwaseong-si, South Korea).

Flap tension was measured via a single-interrupted suture (Prolene 5 − 0, Ethicon, New Jersey, USA) placed at the mid-crestal aspect of the flap using a spring dynamometer (Präzisionsdynamometer, 3B Scientific GmbH, Hamburg, Germany). Flap advancement was also recorded using a periodontal probe (PCPUNC15 probe, Hu-Friedy, Chicago, Illinois, United States). Then, primary wound closure was carried out by a blinded surgeon (EAC), using one horizontal mattress suture and six single-interrupted sutures (Prolene 5 − 0, Ethicon, New Jersey, USA). A second CBCT scan was acquired after closure. Finally, graft materials were rinsed off with water in preparation for the second procedure.

#### Major flap advancement (FA2)

In this group, additional periosteal-releasing incisions and stretching were performed along the same incision line achieving 9–10 mm of flap advancement. The same augmentation protocol, membrane fixation (if allocated), flap tension measurement, primary wound closure, and CBCT scans acquisitions as described in FA1 were conducted.

### Radiographic measurements

**Fig. 2:**
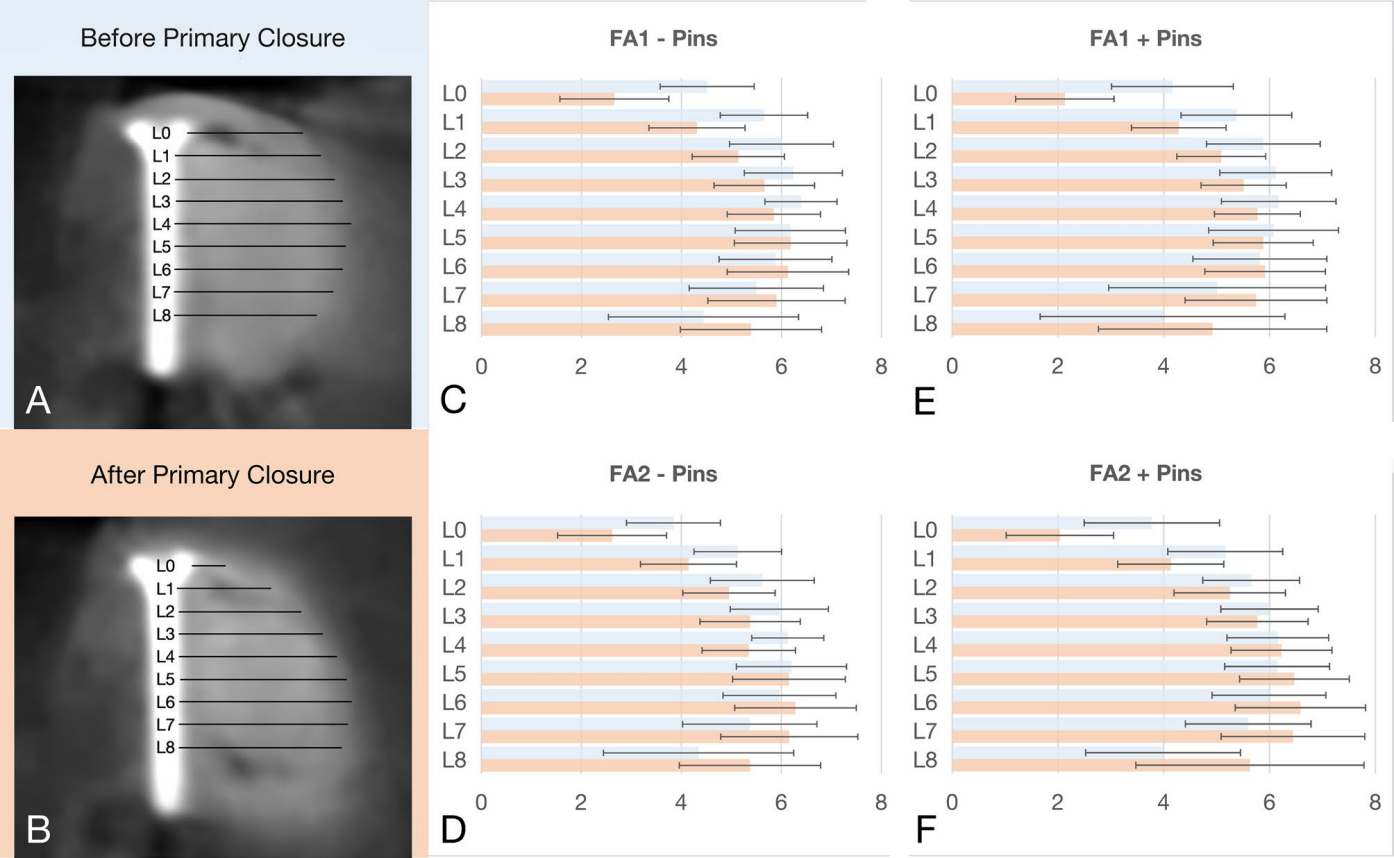
Representative cross-sectional CBCT images from the FA1 - pins group before (**A**) and after primary wound closure (**B**) including the measurements at different apico-crestal levels (L0-L8). Bar plots (± SD) displaying the mean GMT at different levels (L0-L8) for FA1 - Pins (**C**), FA2 - Pins (**D**), FA1 + Pins (**E**) and FA2 + Pins (**F**) before and after primary closure. FA1: Moderate flap advancement. FA2: Major flap advancement

The Digital Imaging and Communications in Medicine (DICOM) files from the acquired CBCT scans were analyzed by an experienced examiner (WZ) using a specialized software (Byzz nxt, version 10.2.121, Orangedental, Biberach, Germany). Cross-sectional images were oriented perpendicularly to both the mandibular arch and the central axis of the fixation screw to ensure consistency. Horizontal graft material thickness (GMT), including both the bone substitute and membrane, was measured perpendicular to the screw surface at 1 mm increments from the platform at the crest level (L0) down to 8 mm (L8) apically. Measurements were taken before (GMT_1) and after primary wound closure (GMT_2) (Fig. 2). After a period of one month, the measurements were repeated by the same examiner.

### Sample size calculation

The sample size was calculated based on the effect size observed in a previous preclinical study with similar test and control measures [18]. A sample size of 15 per group was determined to provide 80% statistical power to detect a mean difference of 0.6 with a standard deviation of 0.5 for both groups and a significance level (alpha) of 0.05 using ANOVA.

### Statistical analysis

The primary outcome was defined as the change in graft material thickness (ΔGMT, %) at platform level L0, before and after primary wound closure. All GMT measurements were obtained by the same examiner in two independent sessions to estimate intra-examiner reliability using the intra-class correlation coefficient (ICC) [24]. The mean measure was then employed for both descriptive and inferential purposes. Descriptive statistics were computed for GMT. Normality was assessed and confirmed using the Kolmogorov-Smirnov test. Paired t-tests were applied to assess ΔGMT before and after wound closure.

To assess the differences in mean ΔGMT changes across FA, fixation, and apico-coronal levels (L0-L8), a general linear model of repeated measures ANOVA was estimated. Since Mauchly’s sphericity hypothesis was rejected, Greenhouse-Geisser correction was employed to estimate the main effects and interactions between factors. Bonferroni’s test was utilized to control for type I errors during multiple comparisons.

To explore correlation, ΔGMT changes and flap advancement were analyzed in relation to the type of fixation and soft tissue phenotypical characteristics using linear regression models estimated via generalized estimation equations (GEE), reporting beta coefficients with 95% confidence intervals (CI). Significant predictors (*p* < 0.05) and marginally significant predictors (*p* < 0.1) were included in a multiple model with interaction terms.

## Results

A total of 60 HBA procedures were conducted on 30 pig hemimandibles and evenly allocated across four experimental groups: FA1 - Pin, FA1 + Pin, FA2 - Pin, and FA2 + Pin (*n* = 15 per group). The intra-examiner consistency for linear CBCT measurements was high, depicting an ICC = 0.85.

### Flap advancement and membrane stabilization

In the FA1 group, the initial periosteal scoring resulted in a mean flap advancement of 4.42 ± 0.67 mm and a mean flap tension of 0.09 ± 0.02 N. In contrast, FA2 group achieved a mean flap advancement of 9.20 ± 0.55 mm and a markedly lower mean flap tension of 0.02 ± 0.01 N. Crestal graft material displacement was significantly reduced in FA2 compared to FA1, with an overall mean ΔGMT at L0 of −35.66 ± 30.68% for FA2 vs. −44.99 ± 20.80% for FA1 (*p* < 0.001). At L4, the overall mean ΔGMT was also significantly lower for FA2 compared to FA1, being − 1.68 ± 13.20% for FA2 vs.−7.20 ± 8.61% for FA1 (*p* = 0.02). No significant differences were observed at the remaining measurement levels (L1–3, L5–8; all *p* > 0.05) (Table 1). Membrane stabilization using fixation pins revealed no significant differences in the overall mean ΔGMTs between pins vs. no-pins (*p* ≥ 0.19) at any measurement level.

**Table 1:** Descriptive statistics (mean ± standard deviation) of the flap advancement (FA1, FA2) and modalities of membrane stabilization (Pins, no PS) at different apico-crestal levels (L0-L8)

Group	Level	GMT_1 (mm)	GMT_2 (mm)	ΔGMT (mm)	ΔGMT (%)
FA1 (FA1 - Pins, FA1 + Pins)	L0	4.26 ± 1.09	2.35 ± 1.01	−1.91 ± 0.96	−44.99 ± 20.80
	L1	5.51 ± 0.96	4.30 ± 0.91	−1.22 ± 0.69	−21.79 ± 11.98
	L2	5.97 ± 0.97	5.11 ± 0.87	−0.86 ± 0.61	−14.04 ± 9.19
	L3	6.18 ± 1.00	5.58 ± 0.90	−0.59 ± 0.57	−9.24 ± 8.33
	L4	6.28 ± 0.91	5.81 ± 0.86	−0.47 ± 0.57	−7.20 ± 8.61
	L5	6.13 ± 1.15	6.03 ± 1.03	−0.10 ± 0.65	−0.69 ± 11.28
	L6	5.85 ± 1.18	6.02 ± 1.16	0.18 ± 0.60	3.88 ± 12.74
	L7	5.25 ± 1.72	5.82 ± 1.34	0.57 ± 1.03	11.10 ± 21.66
	L8	4.21 ± 2.09	5.16 ± 1.81	0.95 ± 1.56	18.77 ± 31.37
FA2 (FA2 - Pins, FA2 + Pins)	L0	3.79 ± 1.22	2.28 ± 1.18	−1.51 ± 1.29	−35.66 ± 30.68
	L1	5.15 ± 1.06	4.14 ± 1.03	−1.01 ± 0.94	−18.40 ± 17.86
	L2	5.64 ± 0.95	5.10 ± 1.15	−0.54 ± 0.94	−9.09 ± 16.87
	L3	5.98 ± 0.94	5.57 ± 1.12	−0.41 ± 0.82	−6.55 ± 14.24
	L4	6.14 ± 0.97	6.02 ± 1.13	−0.12 ± 0.77	−1.68 ± 13.20
	L5	6.17 ± 0.99	6.31 ± 1.18	0.14 ± 0.88	2.85 ± 15.29
	L6	5.98 ± 1.03	6.43 ± 1.29	0.46 ± 1.01	8.61 ± 18.29
	L7	5.48 ± 1.48	6.30 ± 1.46	0.82 ± 1.34	12.40 ± 19.61
	L8	4.21 ± 2.28	5.50 ± 2.04	1.29 ± 2.09	13.92 ± 38.25
No Pins (FA1 - Pins, FA2 - Pins)	L0	4.18 ± 1.13	2.64 ± 1.11	−1.54 ± 0.97	−36.47 ± 23.80
	L1	5.39 ± 0.99	4.23 ± 1.01	−1.16 ± 0.74	−21.35 ± 13.91
	L2	5.84 ± 0.96	5.05 ± 1.09	−0.79 ± 0.73	−13.56 ± 13.05
	L3	6.10 ± 0.98	5.52 ± 1.13	−0.58 ± 0.70	−9.54 ± 12.07
	L4	6.26 ± 0.88	5.83 ± 1.10	−0.43 ± 0.78	−6.69 ± 13.11
	L5	6.19 ± 1.05	6.17 ± 1.21	−0.02 ± 0.95	−0.52 ± 16.86
	L6	5.92 ± 1.06	6.21 ± 1.28	0.29 ± 1.02	6.03 ± 19.68
	L7	5.43 ± 1.54	6.03 ± 1.47	0.60 ± 1.33	8.53 ± 20.20
	L8	4.39 ± 2.23	5.38 ± 1.69	0.99 ± 1.73	12.93 ± 26.91
Pins (FA1 + Pins, FA2 + Pins)	L0	3.97 ± 1.21	2.08 ± 0.96	−1.89 ± 1.24	−44.19 ± 27.85
	L1	5.27 ± 1.05	4.21 ± 0.94	−1.06 ± 0.92	−18.84 ± 16.01
	L2	5.77 ± 0.99	5.17 ± 0.94	−0.60 ± 0.87	−9.56 ± 13.80
	L3	6.06 ± 0.98	5.64 ± 0.88	−0.42 ± 0.72	−6.24 ± 10.75
	L4	6.17 ± 1.01	6.00 ± 0.90	−0.17 ± 0.58	−2.18 ± 8.55
	L5	6.11 ± 1.10	6.18 ± 1.02	0.07 ± 0.55	1.64 ± 8.40
	L6	5.90 ± 1.16	6.25 ± 1.21	0.35 ± 0.61	6.47 ± 10.20
	L7	5.30 ± 1.67	6.09 ± 1.37	0.79 ± 1.05	14.97 ± 20.63
	L8	4.03 ± 2.13	5.28 ± 2.15	1.25 ± 1.95	24.74 ± 34.08
FA1 - Pins	L0	4.51 ± 0.94	2.66 ± 1.09	−1.86 ± 0.70	−42.3 ± 16.5
	L1	5.65 ± 0.88	4.31 ± 0.96	−1.34 ± 0.49	−24.1 ± 9.14
	L2	6.06 ± 0.88	5.14 ± 0.92	−0.92 ± 0.42	−15.3 ± 6.6
	L3	6.24 ± 0.98	5.66 ± 1.00	−0.58 ± 0.56	−9.2 ± 8.2
	L4	6.39 ± 0.72	5.85 ± 0.93	−0.54 ± 0.65	−8.5 ± 9.9
	L5	6.18 ± 1.10	6.18 ± 1.13	0.01 ± 0.79	0.9 ± 14.2
	L6	5.88 ± 1.13	6.13 ± 1.22	0.25 ± 0.74	5.1 ± 16.2
	L7	5.50 ± 1.34	5.90 ± 1.37	0.40 ± 0.78	8.7 ± 18.0
	L8	4.44 ± 1.90	5.39 ± 1.41	0.95 ± 1.69	15.9 ± 25.2
FA1 + Pins	L0	4.16 ± 1.15	2.13 ± 0.93	−2.03 ± 1.12	−47.6 ± 24.6
	L1	5.37 ± 1.05	4.28 ± 0.89	−1.09 ± 0.85	−19.5 ± 14.2
	L2	5.88 ± 1.08	5.09 ± 0.84	−0.80 ± 0.76	−12.7 ± 11.2
	L3	6.12 ± 1.06	5.51 ± 0.81	−0.61 ± 0.61	−9.2 ± 8.6
	L4	6.17 ± 1.08	5.77 ± 0.81	−0.40 ± 0.49	−5.9 ± 7.1
	L5	6.08 ± 1.23	5.88 ± 0.95	−0.20 ± 0.47	−2.3 ± 7.4
	L6	5.82 ± 1.27	5.92 ± 1.14	0.10 ± 0.44	2.5 ± 8.3
	L7	5.01 ± 2.05	5.74 ± 1.34	0.73 ± 1.24	13.5 ± 26.0
	L8	3.98 ± 2.31	4.92 ± 2.16	0.95 ± 1.47	21.7 ± 38.7
FA2 - Pins	L0	3.84 ± 1.23	2.62 ± 1.16	−1.23 ± 1.12	−30.6 ± 29.4
	L1	5.13 ± 1.06	4.14 ± 1.09	−0.99 ± 0.90	−18.6 ± 17.7
	L2	5.62 ± 1.02	4.95 ± 1.26	−0.67 ± 0.94	−11.7 ± 17.7
	L3	5.96 ± 0.99	5.38 ± 1.26	−0.58 ± 0.84	−9.8 ± 15.6
	L4	6.13 ± 1.02	5.81 ± 1.28	−0.32 ± 0.89	−4.8 ± 16.2
	L5	6.20 ± 1.02	6.15 ± 1.33	−0.05 ± 1.12	−2.3 ± 7.4
	L6	5.96 ± 1.03	6.28 ± 1.38	0.32 ± 1.27	6.8 ± 23.7
	L7	5.37 ± 1.75	6.16 ± 1.60	0.79 ± 1.73	8.2 ± 23.6
	L8	4.35 ± 2.59	5.37 ± 1.97	1.03 ± 1.83	9.3 ± 30.4
FA2 + Pins	L0	3.78 ± 1.28	2.04 ± 1.01	−1.74 ± 1.37	−40.7 ± 32.0
	L1	5.16 ± 1.09	4.13 ± 1.00	−1.03 ± 1.01	−18.2 ± 18.5
	L2	5.65 ± 0.91	5.25 ± 1.05	−0.41 ± 0.96	−6.4 ± 16.1
	L3	6.00 ± 0.92	5.77 ± 0.96	−0.23 ± 0.80	−3.2 ± 12.3
	L4	6.16 ± 0.96	6.23 ± 0.95	0.07 ± 0.58	1.5 ± 8.7
	L5	6.14 ± 0.99	6.47 ± 1.04	0.33 ± 0.52	5.5 ± 7.8
	L6	5.99 ± 1.08	6.58 ± 1.23	0.59 ± 0.67	10.3 ± 10.9
	L7	5.60 ± 1.19	6.44 ± 1.36	0.85 ± 0.85	16.2 ± 15.6
	L8	4.08 ± 2.00	5.63 ± 2.16	1.55 ± 2.35	18.1 ± 46.4

*GMT_1* Graft material thickness before primary wound closure, *GMT_2* Graft material thickness after primary wound closure, *ΔGMT* Change in graft material thickness before and after primary wound closure, *FA1* Moderate Flap Advancement, *FA2* Major Flap Advancement

### Interaction term analysis

Within samples applying the same FA, no significant differences were observed regarding mean ΔGMTs between Pins and no-Pins groups across all measurement levels (FA1 + Pins versus FA1 - Pins, *p* ≥ 0.22, and FA2 + Pins versus FA2 - Pins, *p* ≥ 0.22).

In the absence of pins fixation, the mean ΔGMT at the implant platform level (L0) were − 42.3 ± 16.5% and − 30.6 ± 29.4% for FA1 and FA2 (*p* = 0.02), respectively. When pin fixation was applied, the mean ΔGMT were − 47.6 ± 24.6% and − 40.7 ± 32.0% for FA1 and FA2 (*p* = 0.25), respectively.

The correlation analyses for the main effects and interactions revealed that ΔGMT differed significantly depending on the amount of flap advancement (*p* = 0.01), which remained consistent regardless of fixation type (*p* = 0.56), measurement level (*p* = 0.99), and the combination of fixation and level (*p* = 0.56) (Table 2).

**Table 2:** ΔGMT by tension, fixation, and level: results from repeated-measures ANOVA F-test with Greenhouse-Geisser correction for main effects and interactions. FA: Flap advancement

	F value	*p*-value
FA	9.41	0.008**
Fixation	0.32	0.58
Level	53.4	< 0.001***
FA × Fixation	0.36	0.56
FA × Level	0.23	0.99
Fixation × Level	0.42	0.91
FA × Fixation × Level	0.85	0.56

**p < 0.01; ***p < 0.001

### Phenotypic characteristics

The mean KMW at the study sites was 1.93 ± 0.47 mm, and a mean MT3, MT6, MT9 of 1.85 ± 0.49 mm, 3.60 ± 0.99 mm, and 6.15 ± 0.87 mm were recorded, respectively. ΔGMT showed a significant correlation with MT3 (*p* < 0.01) and MT9 (*p* = 0.01), whilst no statistically significant difference was noted for KMW (*p* = 0.77) and MT6 (*p* = 0.15). However, when ΔGMT was adjusted for flap tension, tension remained the most significant predictor of graft displacement changes (*p* < 0.01), while MT3 and MT9 were not considered significant factors influencing ΔGMT (MT3: *p* = 0.06, MT9: *p* = 0.06) (Table 3).

**Table 3:** ΔGMT by phenotypical characteristics. Results of multiple linear regression with GEE model Estimation

	Beta coefficient	95% CI	*p*-value
FA1	0		
FA2	0.46	0.15 0.77	0.004**
MT3	0.51	−0.02 0.04	0.06
MT9	0.31	−0.02 0.64	0.06

**p < 0.01. FA1: Moderate flap advancement FA2: Major flap advancement MT3/MT9: Mucosal thickness at 3 and 9 mm apically from the mucosal margin

## Discussion

This study investigated the influence of flap advancement and membrane stabilization on the dimensional changes of graft materials in uncontained bone defects during HBA. The findings suggest that the degree of flap advancement significantly affects graft material displacement, especially in the crestal area, whereas membrane fixation using two pins did not provide a statistically significant improvement in graft stability. Additionally, the phenotypical soft tissue characteristics did not influence graft displacement. Therefore, H01 was rejected, while H02 and H03 were not.

Achieving primary, passive, tension-free wound closure is a fundamental requirement in most cases for successful HBA procedures [8, 15], as excessive flap tension has been associated with wound dehiscence and potential leading to failure of the augmentation [15, 17]. In the present study, a mucoperiosteal flap with a 0.5 mm periosteal releasing incision enabled moderate flap advancement of 4.42 ± 0.67 mm under a relatively low mean tension of 0.09 ± 0.02 N. Additional periosteal scoring and stretching allowed for major flap advancement (9.20 ± 0.55 mm) with lower tension (0.02 ± 0.01 N). The extent of achieved flap advancement might vary, based on the type of surgical approach, including factors such as the number and design of vertical releasing incisions, flap designs, and the specific method of periosteal scoring applied, among others [10, 25]. Nevertheless, insufficient flap advancement resulting in flap tensions exceeding 0.1 N has been shown to significantly increase the risk of postoperative wound dehiscences, and thereby compromising treatment outcomes [16]. Although currently there is no standardized method to quantify intraoperative flap tension, several approaches have been proposed. For instance, flap tension can be indirectly assessed using tension gauges or digital force measurement devices during flap advancement or suturing [16, 26]. Incorporating such measurements into clinical protocols could help determine the extent of flap advancement needed to achieve tension-free closure, potentially improving the predictability of HBA outcomes.

Preclinical models, such as pig and canine mandibles with pre-post CBCT, are commonly used to evaluate graft stability in HBA [18–21, 26–28]. Previous studies focused on membrane fixation and graft material properties, primarily in box-shaped defects. In contrast, our study highlights the impact of flap tension in a common, yet less frequently investigated defect configuration. In the present study, major flap advancement significantly reduced graft material displacement, compared to moderate flap advancement. Specifically, in the crestal area, the mean ΔGMT was − 35.66% ± 30.68% with major advancement versus − 44.99% ± 20.80% with moderate flap advancement. This suggests that greater flap mobility reduces compressive forces on the graft material, contributing to better preservation of its volume. However, clinicians must also consider the potential drawbacks of excessive periosteal scoring, including compromised local vascularity, increased risk of flap perforation, and reduction of vestibular depth [15].

Different factors can influence graft material displacement, such as the type of bone substitutes and barriers used [18–20, 26]. While various types of graft stabilization approaches have been proposed in the literature, the need for membrane stabilization might largely depend on the defect configuration and remains a topic of debate. In this study, membrane fixation using two pins at the base of the membrane did not significantly alter graft displacement, suggesting that this approach may be insufficient in uncontained defects. This is in line with the findings from a canine model study, which revealed no additional benefits from pin fixation in HBA compared to procedures without pins [27, 28]. Contrarily, a clinical study utilizing titanium pins demonstrated significantly improved volume stability in HBA [22]. Similarly, previous cadaveric studies have shown reduced membrane collapse and enhanced graft stability when two bone pins were used in contained defects [18, 19]. Based on these findings, the present study applied the same fixation strategy to uncontained defect morphologies to evaluate its performance under more challenging conditions. These discrepancies observed can be attributed to variations in defect morphology and the position and number of fixation pins used across studies. Indeed, bone defect morphology plays a critical role in the success of HBA [29–31], with most studies to date investigating box-shaped (contained) bone defects [18, 19, 21, 27]. This contained defect morphology may inherently be more favorable in limiting bone grafting materials displacement offering structural support by the surrounding bony walls [26, 29]. In contrast, uncontained defects, such as the horizontal concavities modeled in this study, lack the structural support given by bony walls, thus predisposing to greater graft displacement. In addition to morphology, pig mandibular defect studies have highlighted the influence of different graft material combinations on volume stability during primary wound closure. In these, the use of bone blocks or non-resorbable membranes presented superior volume stability, in comparison to particulate bone grafts or collagen membranes during primary closure. Therefore, these findings underline the importance of developing defect-specific guidelines for the selection of appropriate biomaterials, barrier membranes and stabilization techniques [18–20, 26].

Regarding soft tissue phenotypical characteristics, the present study found no significant influence of KMW and MT on graft displacement. This is in line with a previous preclinical study, in which the soft tissue parameters did not affect the extent of flap advancement or graft displacement across different surgical flap techniques [21]. However, clinical evidence suggests that soft tissue phenotypical characteristics such as thin mucosa phenotype, lack of KMW, or the presence of scar tissue may increase the risk of postoperative complications after HBA. In such cases, it has been proposed that pre-surgical or simultaneous soft tissue augmentation could enhance HBA outcomes by improving mucosal resilience and wound healing [32, 33]. Notably, Burkhardt and Lang demonstrated that MT (≤ 1 mm vs. >1 mm) only influenced the risk of postoperative wound dehiscence when flap tensions exceeded 0.10 N [16], reinforcing the notion that flap tension as the primary determinant of uneventful wound healing, with this phenotypic feature acting as a protective factor.

The present study introduces a clinically relevant model featuring a pronounced horizontal alveolar ridge deficiency with an uncontained defect morphology, a common real-world scenario in implant dentistry that frequently is associated with buccal peri-implant dehiscence-type defects and the need for HBA. However, when interpreting the results of the present study, several limitations must be taken into account. The dimensional stability of graft materials was assessed immediately after primary wound closure, without a healing period. Due to the ex vivo design, it was not possible to replicate multiple relevant clinical variables that may influence graft displacement, such as postoperative inflammation, swelling, wound remodeling, and tissue movement during mastication, speech, or patient manipulation. Additionally, only one specific defect morphology and stabilization method were assessed, leaving the effect of different defect configurations unclear. The potential influence of alternative stabilization approaches, such as increasing the number of pins, periosteal suturing, or the use of fibrin sealant, was not assessed and warrants further investigation. Performing two flap advancement procedures on the same hemimandible may have introduced soft tissue fatigue or reduced elasticity over time. Nevertheless, continuous hydration with sterile saline helped to preserve tissue integrity. Otherwise, the use of the same site ensured consistent soft tissue phenotype and defect morphology between procedures, thereby reducing variability and isolating the effect of flap advancement. Finally, all radiographic measurements were performed by a single calibrated examiner. Although intra-rater reliability was high, the lack of a second blinded examiner may limit the objectivity of the measurements and introduce measurement bias.

Further in vivo animal studies or human clinical trials are needed to confirm these findings under biologically active conditions, especially to assess soft tissue healing, graft integration, and long-term outcomes. Additionally, incorporating digital simulations or biomechanical modeling potentially could allow for visualizing real-time graft deformation under tissue movement.

## Conclusion

Greater flap advancement and reduced flap tension were associated with significantly less graft material displacement, while membrane fixation with two pins alone proved insufficient to ensure graft stability in challenging, uncontained defect morphologies.

## Data Availability

The datasets used and analyzed during the current study are available from the corresponding author on reasonable request.

## Abbreviations

ANOVA: Analysis of variance
CBCT: Cone-beam computed tomography
CI: Confidence intervals
DICOM: Digital imaging and communications in medicine
FA: Flap advancement
GEE: Generalized estimation equations
GBR: Guided bone regeneration
HBA: Horizontal bone augmentation
ICC: Intraclass correlation coefficient
KMW: Keratinized mucosa width
L: Level
MT: Mucosal thickness
